# Constructing diplomatic discourse: a corpus-driven analysis of the discursive strategies by the spokespersons of China's ministry of foreign affairs during a public health crisis

**DOI:** 10.3389/fpsyg.2025.1635767

**Published:** 2025-08-05

**Authors:** Tao Li, Feng Pan

**Affiliations:** ^1^Centre for Corpus Research, Shanghai Ocean University, Shanghai, China; ^2^Shanghai International Studies University, Shanghai, China

**Keywords:** press conference, discursive strategy, spokesperson, diplomatic discourse, public health crisis

## Abstract

Drawing on a corpus-driven discourse analysis approach, this paper examines the discursive strategies adopted by the spokespersons of China's Ministry of Foreign Affairs during regular press conferences regarding a public health crisis. The analysis reveals that (1) the spokespersons actively used communicative discursive strategies to articulate China's stance and international cooperation initiatives while also employing offensive discursive strategies to counter criticisms from Western countries and media regarding the virus and the pandemic; (2) Interestingly, a juxtapositional discursive strategy was observed, by which China, together with other countries, was represented as the positive majority, whereas the US and the UK's media BBC as the negative minority, reinforcing the rationality of China's policies. It is argued that the spokespersons' use of discursive strategies can be attributed to China's geopolitical dynamics with the West and the influence of traditional Chinese culture on China's diplomatic policies.

## 1 Introduction

There has been growing interest in examining the features of spokespersons' discourse in recent years (Marakhovskaiia and Partington, 2019; Mao and Zhao, 2020; Liu, 2022; Wu, 2023; Zhang and Tang, 2024; Zhang et al., 2025). For example, Wu (2021) analyzed the argumentative styles used in the discourse of the spokespersons of China's Ministry of Foreign Affairs (CMFA), revealing that a confrontational style was frequently adopted when addressing critics of China or those holding views considered unacceptable by the Chinese government. Parallel to this, Cai and Xu (2023) conducted an analysis of 126 texts from CMFA's regular press conferences focusing on the origin tracing of COVID-19. Their findings indicate that the spokespersons predominantly employed a “seeking similarity” discursive strategy, often by adeptly citing authoritative sources like scientists and the World Health Organization to persuade international audiences to align with China's position. Examining COVID-19-related tweets from the CMFA spokespersons, Wu and Feng (2023) also found that offensive strategies were utilized to rebut US accusations.

However, previous research on CMFA spokespersons' discourse has mainly focused on the features of their discursive strategies, with limited attention to the factors shaping these characteristics, especially during COVID-19 and the period when they were labeled as “wolf warrior diplomats” in Western politics and media (Martin, 2021; Huang, 2022; Dai and Luqiu, 2022). Moreover, studies are also needed on how particular social reality is discursively represented internationally by the spokespersons in an era when China proactively strives for discursive power in the international arena.

Outbreaking in late 2019, COVID-19 quickly became a global health crisis, generating a multitude of discourses characterized by different voices (Breeze, 2021; Jaworsky and Qiaoan, 2021; Breeze and Gintsburg, 2023; Chan and Yu, 2023; Zhang, 2024; Heimo et al., 2025; Murry, 2025). Particularly, the discursive controversy surrounding COVID-19 between China and the West has led to extensive discussions and research. For example, Pietrzak-Franger et al. (2022) found that stigmatization strategies are skillfully used to blame China for COVID-19 in all the three Western newspapers (the UK, Germany, and Austria). They also argued that China was targeted as a scapegoat in narrating the pandemic mainly because of the West's anxieties over China's potential rise to world dominance. Phillips and Cassidy (2024) examined the ways in which COVID-19 was discursively constructed by the media from China and the UK and found that British media adopted a pessimistic and sensational tone by accenting the virus' unknown nature, whereas Chinese reports presented a more optimistic tone by highlighting local success. Though the data were retrieved from the Nexis database, the results rely on a qualitative analysis of only 12 news reports. While these studies are based on a limited amount of data, their results highlight the dialectical relationship between language use and social reality, demonstrating that discourse not only reflects but also shapes social reality.

Discourse on COVID-19 has been produced in other various contexts apart from news media, such as speeches by governmental leaders, think tank reports, and press conferences. Particularly in press conferences, spokespersons for a country's Ministry of Foreign Affairs use discursive strategies to articulate their nation's stance, shaping international perceptions. Cai and Xu (2023, p. 479) argue that the regular press conferences of CMFA are “an authoritative channel for the world to understand China's voice and a frontline for constructing China's discursive power in diplomacy”. Thus, examining the CMFA spokespersons' discourse provides valuable insights into the discursive strategies frequently employed by diplomatic spokespersons to construct national narratives and convey official stances.

This paper aims to examine the discursive strategies adopted by the CMFA spokespersons with a case study of their discourse during regular press conferences regarding COVID-19. Specifically, it addresses the following questions: (1) How did the spokespersons discursively represent “病毒” (virus) and “疫情” (pandemic), two typical lexical items related to COVID-19, in the regular press conferences of CMFA? (2) what discursive strategies were used by CMFA spokespersons in the discursive context of COVID-19? (3) What are the factors, institutional or social, that underpin the spokespersons' choice of different discursive strategies?

## 2 Corpus-driven method and data collection

A corpus-driven method was adopted to achieve the stated objectives. This methodological approach follows a path where “observation leads to hypothesis leads to generalization leads to unification in theoretical statement” (Tognini-Bonelli, 2001, p. 85). It emphasizes an inductive analysis of linguistic patterns emerging directly from the corpus data/discourse, rather than relying on prior theoretical frameworks. It allows categories, collocations, and discourse patterns to be identified empirically through systematic observation of the corpus itself. This bottom-up method is particularly suited for exploring unfamiliar or evolving discursive practices—such as those used by government spokespersons during times of crisis—where new discursive strategies may emerge.

The corpus under investigation comprises transcripts of CMFA's regular press briefings from January 2020 to December 2021, the first 2 years of COVID-19. All corpus data are publicly available at https://www.fmprc.gov.cn/eng/xw/fyrbt/. Two considerations account for the selection of these data. Firstly, COVID-19 exerted a far-reaching impact, with global debates and discussions on the pandemic emerging as the focus of international attention since its outbreak. Secondly, amid unprecedented global changes, some studies have shown that the CMFA spokespersons grew increasingly aggressive in their discourse and were described as “wolf warrior diplomats” (Martin, 2021; Huang, 2022; Dai and Luqiu, 2022). It is thus worthwhile to examine the actual characteristics of this particular discourse by CMFA spokespersons within the diplomatic context of press conferences.

The corpus data excludes journalists' questions and retains only the spokespersons' responses, since the study focuses on their discourse. For data retrieval, all Chinese response data was segmented using SegmentAnt_jieba, an essential step because Chinese characters have no space in-between, making them incompatible with corpus processing tools like WordSmith, AntConc. After auto-segmentation, we also conducted a manual check to ensure accuracy. The final CMFA spokespersons' responses corpus consists of 782,593 Chinese words. For generating collocates, WordSmith (8.0) was selected for its capacity to compute collocational relationships through statistically comparing with the corpus under investigation and a reference corpus, using measures such as MI^3^. Torch2019, a balanced corpus containing one million Chinese words, was used as the reference corpus for these calculations.

## 3 Discursive representation of “病毒”(Virus) and “疫情”(Pandemic)

Using WordSmith (8.0), we extracted all concordance lines for the search items “病毒” and “疫情” respectively. As “discourse is also constructed through collocations” (Gu, 2019), we also generated collocate lists for both items within a five-word span (left and right of the search items). The collocates of the two search items were then ranked respectively in descending order based on the MI^3^ scores, and their frequencies were marked. Based on the context of the concordance lines, all collocates for “病毒” and “疫情” were categorized according to their thematic properties.

### 3.1 Discursive representation of “病毒”(virus)

Of the 59 collocates generated for “病毒”, we manually removed four collocates related to question sequences and time (“第一个”[first), “第二个”[second), “下半年”[second half of the year), “近年来”[in recent years]), resulting in 55 remaining collocates. Thematically, these 55 collocates were classified into five categories as detailed in Table 1.

**Table 1:** The collocates of “病毒” (virus) in the CMFA spokespersons' responses.

**Category**	**Collocates**	**Frequency and ratio**
Virus origin and research	实验室 (laboratory, *f* = 97); 研究所 (research institute, *f* = 97); 专家组 (expert group, *f* = 16); 科学家 (scientist, *f* = 16); 基因组 (genome, *f* = 9); 科学研究 (scientific research, *f* = 9); 起源于 (originated from, *f* = 5); 自然界 (nature, *f* = 4); 湖北省 (Hubei Province, *f* = 6); 武汉市 (Wuhan City, *f* = 6); 吹风会 (press briefing, *f* = 3); 搞清楚 (clarify, *f* = 2); 科研工作 (scientific research work, *f* = 2); 马里兰州 (Maryland, *f* = 2); 普遍认为 (It is widely believed that, there is no evidence that the new coronavirus originated from a laboratory, *f* =2); 卫生部 (Department of Health, *f* = 2); 事实证明 (所谓中国散播病毒纯属无稽之谈) (facts prove that, it is a completely groundless claim that China spread the virus, *f* = 2); 有没有 (产生新冠病毒) (Is there novel coronavirus originated?, *f* = 3); (详细列举了新冠病毒在美暴发的) 时间表 (detailed schedule of, the COVID-19 outbreak in the US, *f* = 2); 发布会 (press conference, *f* = 3); 研究成果 (research results, *f* = 2); 传染病 (infectious disease, *f* = 2); 安德森 (Anderson, *f* = 2); 科学界 (scientific community, *f* = 2); 负责人 (person in charge, *f* = 4); 中国科学院 (Chinese Academy of Sciences, *f* = 3); (不) 意味着 (武汉就是新冠病毒的源头) (doesn't, mean, Wuhan is the origin of the novel coronavirus, *f* = 4); 中科院 (CAS, *f* = 2); 可能性 (possibility, *f* = 3); (赤道几内亚新冠病毒应对与监测) 委员会 (committee, for response and monitoring of COVID-19 in Equatorial Guinea, *f* = 4); 工作人员 (staff, *f* = 3); 工作人员 (researcher, *f* = 2); 实际上 (in fact, the virus originated in Le Roy, Kansas, USA, *f* = 3); 越来越 (多关于病毒和疫情2019年下半年就已在世界多地多点出现的报道) (More and more reports about the virus and the pandemic have appeared around the world since the second half of 2019, *f* = 4); 研究所 (research institute, *f* = 2); 事实上 (In fact, there have been too many conspiracy theories regarding the origin of the virus directed at China, *f* = 2)	332; 74.77%
Virus impact	全人类 (all humanity, *f* = 12); 受害者 (victims, *f* = 7); 全世界 (all over the world, *f* = 7); 种族主义(racism, *f* = 4); 外交官 (diplomats, *f* = 3); 感染者 (infected individuals, *f* = 2); 前所未见 (unprecedented, *f* = 2); 突如其来 (sudden, *f* = 3); 西班牙 (spain, *f* = 5)	45; 10.14%
Anti-pandemic agents	中国政府 (Chinese government, *f* = 6); (世界卫生组织) 成员国 (member states, of the WHO, *f* = 7)	13; 2.93%
China's attitudes	高度重视 (highly valued, *f* = 3); 作出贡献 (make contributions, *f* = 2); 进一步(研究); (further research, *f* = 3)	8; 1.80%
The US attitudes	政治化(politicization, *f* = 34); 处心积虑 (calculatingly, *f* = 3); 意识形态 (ideology, *f* = 4); 迫不及待 (impatiently, *f* = 2); 为什么 (不依赖科学家而要把情报部门作为主导力量) (Why relying on intelligence agencies rather than scientists as the leading force, *f* = 3)	46; 10.36%

As Table 1 illustrates, the first category, “virus origin and research”, primarily focuses on scientific investigations into the origin of the novel coronavirus, particularly the debate over whether it emerged naturally or via laboratory leakage. This includes discussions on the roles of laboratories and research institutes, the participation of scientists, references to genomes and research findings, and debates on whether the virus originated from the city of Wuhan in Hubei Province. This category of collocates constitutes the highest proportion, accounting for 74.77%. This indicates the spokespersons' primary focus on the issue of virus origin and the attempts by foreign politicians and media to smear China with labels such as “Chinese virus”. Besides, the spokespersons also emphasized the scientific nature of virus tracing, frequently referring to scientific research organizations such as “institutes”, “Chinese Academy of Sciences”, “research academies”, “research community”, and mentioning “scientists” as well as specific researchers like “Anderson”.

The second category, “virus impact”, which addresses the global ramifications of the novel coronavirus, is also a focal point for the spokespersons, accounting for 10.14% of the total. By highlighting the virus' sudden onset, severity, and rapid spread (e.g., “sudden emergence”, “unprecedented intensity”), the spokespersons emphasized its widespread impact on “the entire world” and “all humanity”, including social issues such as “racism”. Notable examples include statements like “Diplomats have immunity due to their posts, but the virus does not know that” (April 3^rd^, 2020).

As the third category shows, the collocates also reveal that the two primary agents engaged in virus combat are the Chinese government and some World Health Organization member states. This indicates that the spokespersons did not foreground other agents involved in the fight against the virus, because few were specifically mentioned apart from China. However, this could also indicate that virus-combating agents were not a priority in their discourse; instead, the emphasis was placed on such aspects as the attitudes and positionings of involved agents toward the virus.

Turning to the fourth category, the analysis reveals a positive representation of China's anti-virus stance, with spokespersons emphasizing that “The Chinese government attaches high importance to COVID-19 vaccine R&D” (January 15^th^, 2021). In contrast, the fifth category indicates a negative representation of the US stance, where the spokespersons criticized that “the US politicization of COVID-19 origins tracing is repeating the history of the ‘Spanish Flu”' (August 17th, 2021). Interestingly, the frequency of negative representations of the US stance far exceeds that of positive representations of China's stance (46 vs. 8).

The pattern identified above aligns with the discursive strategies observed in the spokespersons' tweets regarding COVID-19 (Wu and Feng, 2023), where offensive strategies are favored over defensive ones. For instance, one spokesperson stated, “We hope the US will reflect upon its behaviors, eliminate its political virus of ideological bias, and stop vilifying the CPC and Chinese media” (March 20, 2020), a clear manifestation of such offensive strategies.

### 3.2 Discursive representation of “疫情”(pandemic)

For “疫情”, a total of 190 collocates were generated, and we manually removed seven collocates related to question sequences and time (“第一个”[first], “第二个”[second], “一段时间”[a period of time], “上半年”[first half of the year], “下半年”[second half of the year], “一个月”[one month], “与此同”[meanwhile]). In addition, the collocate “想方设法”(trying every possible means) was grouped into two categories in two different contexts. As a result, 184 collocates were included for analysis, which were classified into six categories thematically as presented in Table 2.

**Table 2:** The collocates of “疫情” (pandemic) in the CMFA spokespersons' responses.

**Category**	**Collocates**	**Frequency and ratio**
Public health (crisis)	公共卫生 (public health, *f* = 51); 专家组 (expert group, *f* = 12); 流行病 (epidemic, *f* = 11); 医护人员 (medical staff, *f* = 7); 中医药 (traditional Chinese medicine, *f* = 7); 身体健康 (physical health, *f* = 6); 发展趋势 (development trends, *f* = 5); 死亡率 (mortality rate, *f* = 5); 流行病学 (epidemiology, *f* = 3); 传染病 (infectious disease, *f* = 3); 实验室 (laboratory, *f* = 5); 医疗卫生 (medical hygiene, *f* = 2); 研究所 (research institute, *f* = 2)	119; 9.54%
Pandemic impact	经济社会 (economic and social, *f* = 43); 突如其来 (sudden, *f* = 27); 全球性 (global, *f* = 14); 新一轮 (new round, *f* = 12); 供应链 (supply chain, *f* = 10); 产业链 (industrial chain, *f* = 9); 全人类 (all humanity, *f* = 8); 台湾同胞 (Taiwan compatriots, *f* = 8); 大变局 (major change, *f* = 4); 深刻影响(profound impact, *f* = 4); (没有任何国家可以) 独善其身 (no country can, stand alone, *f* = 3); (尽快) 恢复正常 (resume normalcy, as soon as possible, *f* = 3); 种族歧视 (racial discrimination, *f* = 3); 无论是 (whether it's, *f* = 4); 武汉市(Wuhan city, *f* = 5); 受害者 (victim, *f* = 4); 热点问题 (hot issue, *f* = 2); 突发事件 (emergency, *f* = 2); 突然袭击 (sudden attack, *f* = 2); 遭遇战 (encounter battle, *f* = 2); 留学生 (international students, *f* = 6); 成千上万 ( 美国人感染/死亡) (thousands upon thousands, American infected/deceased, *f* = 2); 呈现出 ( 叠加态势) (presenting, superimposed trend, *f* = 2); 世界各地 (all over the world, *f* = 2); 种族主义 (small and medium-sized enterprises, *f* = 2); 种族主义 (racism, *f* = 2); 取决于 (疫情进展) (depends on, pandemic progression, *f* = 3); 不确定性 (uncertainty, *f* = 3); 越来越 (more and more, global challenges/people losing life, *f* = 3)	194; 15.56%
China's anti-pandemic efforts and attitudes	中国政府 (Chinese government, *f* = 87); 力所能及 (to the best of one's ability, *f* = 21); 感同身受 (empathize, *f* = 18); 团结一心 (united as one, *f* = 16); 积极开展 (actively carry out, *f* = 15); 众志成城 (forge a common front, *f* = 14); 白皮书 (white paper, *f* = 12); 高度重视 (highly value, *f* = 12); 外交部 (Ministry of Foreign Affairs, *f* = 8); 有把握 (confident, *f* = 8); 不得已 (have no choice but to, *f* = 7); 万众一心 (all the people unite as one, *f* = 6); 全力以赴 (spare no effort, *f* = 9); 短时间 (short period of time, *f* = 5); 法律法规 (laws and regulations, *f* = 5); 国务委员 (state councilor, *f* = 4); 战略性(胜利) (strategic, victory, *f* = 6); 保卫战 (defensive battle, *f* = 4); 人民战争 (people's war, *f* = 3); 展现出(信心)(demonstrate, confidence, *f* = 3); 阶段性(成果) (phasal, results, *f* = 4); 想方设法(最大程度地减少疫情扩散蔓延 (do everything possible, to minimize the spread and transmission of the pandemic to the greatest extent, *f* = 2); 不畏艰险 (fear no difficulties, *f* = 2); 指挥部 (headquarters, *f* = 4); 党和政府 (party and government, *f* = 2); 公开信 (open letter, *f* = 2); 宏观经济 (macroeconomy, *f* = 2); 宏观政策 (macro policy, *f* = 2); 结合实际 (in accordance with the actual situation, *f* = 2); 巨大贡献 (great contribution, *f* = 2); 临时性 (temporary nature, *f* = 2); 领导小组 (leadership group, *f* = 2); 满意度 (satisfaction level, *f* = 2); (绝 不...) 前功尽弃 (never, abandon previous achievements, *f* = 2); 一系列(防疫措施等) (a series of, pandemic prevention measures, *f* = 8); 清清楚楚 (clearly, *f* = 2); (发布)前一天 (疫情信息) (release, pandemic information of, the day before, *f* = 3); 出入境 (entry and exit, *f* = 3); 行之有效 (effective, *f* = 2); 冬奥会 (winter Olympics, *f* = 3); 北京市 北京市(Beijing city, *f* = 3); 各族人民 (people of all ethnic groups, *f* = 2); 负责人 (person in charge, *f* = 4); 强有力 (powerful, *f* = 2); 中国共产党 (Chinese Communist Party, *f* = 4); 得益于 (benefit from, *f* = 2); 大规模 (人道主义) (large-scale, humanitarian, *f* = 3); 发布会 (press conference, *f* = 2); 习近平 (Xi Jinping, *f* = 6); 研讨会 (seminar, *f* = 2); 主管部门 (competent authority, *f* = 2); 工作者 (worker, *f* = 2); 国务院 (State Council, *f* = 3); (不) 意味着 (就是疫情起源的地方) (doesn't, mean, the place of origin of the pandemic, *f* = 2); 奥运会 (Olympic Games, *f* = 2); (领导小组) 办公室 (leadership group, office, *f* = 2)	359; 28.79%
China's engagement in international anti-pandemic cooperation	同舟共济 (we are all in the same boat, *f* = 40); 致力于 (dedicated to, *f* = 7); 相互支持(mutual support, *f* = 26); 关键时刻 (critical moment, *f* = 18); 关键时刻 (the whole world, *f* = 18); 全世界 (top priority, *f* = 9); 当务之急 (work together, *f* = 9); 齐心协力 (make contributions, *f* = 9); 作出贡献 (beneficial to, *f* = 18); 充分肯定 (fully affirm, *f* = 8); 联合声明 (joint statement, *f* = 6); 发挥作用 (play a role, *f* = 5); 来之不易 (hard-won, *f* =6); 第二次 (会议) (second, meeting, *f* = 4); 人道主义 (humanitarian, *f* = 4); 团结一致 (united as one, *f* = 4); 友好合作 (friendly cooperation, *f* = 5); 大力支持(strongly support, *f* = 3); 积极支持(actively support, *f* = 3); 重大胜利 (significant victory, *f* = 3); (各方) 进一步 (all parties, further, *f* = 28); 共同体 (community, *f* = 10); 联席会议 (joint meeting, *f* = 3); 有助于 (conducive to, *f* = 9); 绝大多数 (vast majority, *f* = 6); 至关重要 (vitally important, *f* = 5); 不断加强 (continuously strengthen, *f* = 2); 公平正义 (fairness and justice, *f* = 2); 积极意义 (positive significance, *f* = 2); 通电话(make a phone call, *f* = 2); 新台阶 (new level, *f* = 2); 面对面 (face-to-face, *f* = 3); 有识之士 (knowledgeable person, *f* = 2); 实实在在 (提供给发展中国家的帮助) (substantially, providing assistance to developing countries, *f* = 3); 一如既往 (支持其他国家抗疫)(as always, supporting other countries in pandemic prevention, *f* = 2); (世卫组织作用) 不可或缺 (the role of the WHO, is indispensable, *f* = 2); 综合性 (联大；决议)(comprehensive, un/resolution, *f* = 3); 南南合作 (south-south cooperation, *f* = 2); 高水平 (合作) (high-level, cooperation, *f* = 2); 尽可能 (控制疫情等) (as far as possible, control pandemic, *f* = 2); 什么样 (what kind, *f* = 2); 针对性 (targeted, *f* = 2)	301; 24.14%
Regions and organizations supporting anti-pandemic cooperation	台湾地区 (Taiwan region, *f* = 23); 发展中国家 (developing countries, *f* = 23); 联合国 (United Nations, *f* = 18); 太平洋 (岛国) (Pacific, island nations, *f* = 12); 阿拉伯 (Arab, *f* = 10); 柬埔寨 (Cambodia, *f* = 9); 委内瑞拉 (Venezuela, *f* = 8); 菲律宾 (Philippines, *f* = 7); 阿富汗 (Afghanistan, *f* = 5); 孟加拉国 (Bangladesh, *f* = 5); (乌兹别克斯坦, 意大利等) 卫生部) (health ministries, such as Uzbekistan, Italy, *f* = 5); (世界卫生组织等) 成员国 (member states of, the WHO, *f* = 8); 意大利 (Italy, *f* = 13); 海峡两岸 (the two sides across the Straits, *f* = 4); 安理会 (Security Council, *f* = 3); 尼泊尔 (Nepal, *f* = 3); (东盟、中日韩等)领导人) (leaders, from ASEAN, China, Japan, South Korea, etc., *f* = 11); 加拿大 (Canada, *f* = 7); 安哥拉 (Angola, *f* = 2); (世界卫生组织) 执委会 (the Executive Board, of WHO, *f* = 3); (阿盟、世界卫生组织等)秘书处 (the Secretariats, of the Arab League/the WHO), *f* = 3); (世界卫生组织等) 委员会 (the Committees of the WHO), *f* = 5); 东南亚 (Southeast Asia, *f* = 2); 塞尔维亚 (Serbia, *f* = 2); 伊斯兰 (Islam, *f* = 3); 新加坡 (Singapore, *f* = 2); 俄罗斯 (Russia, *f* = 2)	198; 15.88%
Regions and organizations disturbing anti-pandemic cooperation and their attitudes	(我要敦促美方, 立即停止对疫情) 政治化 (I urge the US to immediately stop politicizing the pandemic, *f* = 29); 美国政府 (the US government, *f* = 5); BBC(BBC, *f* = 4); (美国无端质疑中国在疫情防控问题上的) 透明度 (The US ungrounded questioning of China's transparency in pandemic prevention and control, *f* = 3); (美国行为) 不利于 (国际社会齐心协力抗击疫情) The US acted detrimentally to international solidarity in fighting the pandemic, *f* = 4); (我们奉劝美方……) 想方设法 (地最大程度地维护人民生命安全) (We urge the US to try their best to safeguard the safety of people's lives, *f* = 1); (美国)长时间 (蓄意淡化疫情) (the US for a long time deliberately downplay the pandemic, *f* = 2); 事实证明 (Facts prove, *f* = 2); (美国) 预料到 (一个月后国内的疫情会扩散蔓延) (The US anticipated 1 month later the domestic pandemic will spread, *f* = 2); (美国……与当前国际抗疫合作) 背道而驰 (The US is contrary to current international cooperation in pandemic control, *f* = 2); 凌驾于 (superior to, *f* = 2); (美国 “积极攻击中国”以应对疫情”) 备忘录 (The memorandum of the US “actively attacking China” to deal with the pandemic, *f* = 2); 为什么 (美国三缄其口其与疫情相关的流感等) (Why the US remains silent on its flu and other pandemic-related issues, *f* = 7); 事实上 (in fact, *f* = 4); 实际上 (in reality, *f* = 3); 如果说 (美方在疫情初期受到误导或者没有得到充足的信息) (If the US was misled or did not receive sufficient information in the early stages of the pandemic, *f* = 2); (BBC) 纪录片 (混淆画面指责中国) (BBC documentary mixing images to blame China, *f* = 2)	76; 6.09%

The first category “public health (crisis)” primarily centers on health, medical service, and scientific research related to COVID-19. Particularly, it emphasizes the pivotal role of scientists and medical personnel in pandemic prevention and control, while also mentioning the efficacy of traditional Chinese medicine in COVID-19 prevention. However, the proportion of this category is relatively low, accounting only for less than 10%.

The collocates in the second category “pandemic impact” indicate the profound influence of COVID-19 on the global economy. The sudden outbreak of COVID-19 has triggered a new wave of economic shocks and instability in supply and industrial chains, affecting adversely not only the small and medium-sized enterprises, but also the livelihoods of all people worldwide. For example, “[W]hen tens of thousands of American people are struggling against COVID-19, the two parties are attacking each other ferociously and putting their own political interests above people's life and health” (August 20^th^, 2021). Similar to the collocates in the “virus impact” category as discussed in Section 3.1, those in the “pandemic impact” category also highlight other social issues brought about by COVID-19, such as “racism” and “uncertainty” of the international situation, all of which pave a solid foundation for the formulation of discourse on global cooperation in pandemic prevention.

The collocates in the third category of “China's anti-pandemic efforts and attitudes” are mainly about China's efforts and stance toward pandemic prevention, accounting for the highest proportion of 28.79%. The collocates involve many agents in pandemic prevention from the President Xi Jinping to people of all ethnic groups nationwide, as well as various levels of Party committees and governments. This demonstrates that the entire population highly value pandemic prevention. In this context where combating the pandemic was described as a war that must be won, the Chinese people faced challenges without fear, united as one to make concerted efforts to carry out the defensive battle and ultimately achieved a strategic victory. These collocates strongly praise China's efforts and attitude in pandemic prevention. This also indicates that regarding the collocates of “pandemic”, the spokespersons discursively tended to adopt communicative strategies, aiming to promote “their own concepts, practices, emotions to express China's attitude and position, and to uphold China's good image internationally” (Wu and Feng, 2023, p. 73).

The collocates in the fourth category “China's engagement in international anti-pandemic cooperation” highlight China's collaboration with other international entities in combating COVID-19, and China's attitude toward cooperative efforts in pandemic prevention. The use of these collocates potentially indicates that China was trying to show their dedicated efforts for global pandemic control through high-level cooperation with other countries and international organizations. China also provided substantial assistance and tangible support to developing countries, upholding fairness and justice. Furthermore, China also valued international mechanisms for cooperative pandemic control, supporting the comprehensive roles of institutions such as the United Nations and the World Health Organization and promoting South-South cooperation channels. For instance, statements, like “China will continue to do the right thing, provide assistance to others as its capacity permits, and work with the international community to secure the final victory against the pandemic” (April 22^nd^, 2020), showcase China's commitment to international cooperation in pandemic control.

The fifth category “regions and organizations supporting anti-pandemic cooperation” also reflect, to a certain degree, China's approach and attitude toward pandemic control. The purpose of classifying this category is to compare those countries/regions and organizations that support international cooperation and those that do not. When the frequencies are combined, the total frequency of China and other actively participating entities in pandemic control reaches 660, accounting for 52.93% of the total. This far exceeds the ratio of 6.09% for the US and the BBC, which are shown to disrupt international cooperation. The result further highlights that China's advocacy for international cooperation aligns with international common interests. Furthermore, apart from China as the main participant in international cooperation, there are as many as 27 countries/regions or organizations involved, including major developed Western countries. This is evidenced by statements such as “China and Canada have been cooperating and giving each other valuable support during the difficult fight against COVID-19” (September 18^th^, 2020). In contrast, there are only two entities that are specifically mentioned as disrupting pandemic control, i.e., the US and the BBC. The sharp disparity in numbers again reaffirms that China stands with the majority of the international community dedicated to striving for the good of all humans.

On the one hand, the collocational patterns identified regarding the fifth and the sixth categories demonstrate that China's proactive advocacy for international cooperation in pandemic control had been widely embraced by many countries/regions and organizations, who represent the mainstream of the international community. In contrast, the smears against China's pandemic response by the US and the UK, coupled with their own ineffective pandemic control measures, constituted a minority standpoint globally. On the other hand, the total frequency of collocates representing the 27 entities participating in cooperative efforts is 198, accounting for 15.88% of the total. In contrast, the total frequency of collocates representing the US and the BBC is 76, taking up only 6.09%. If the BBC's frequency of 6 is excluded, the frequency of negative representations attributed to the US reaches as many as 70 times. This indicates that the spokespersons adopted a variety of offensive discursive strategies, rather than the previously adopted passive approach of prioritizing harmony, to counter the US' attempts to discredit China and to criticize the US's own ineffective anti-pandemic response.

To summarize, the CMFA spokespersons used communicative discursive strategies to articulate China's stance and international cooperation initiatives while also employing offensive discursive strategies to counter criticisms from some Western countries and media regarding COVID-19. They also interestingly adopted a juxtapositional discursive strategy by which China, together with other countries, was represented as the positive majority, whereas the US and the UK's media BBC as the negative minority, reinforcing the rationality of China's policies.

As Marakhovskaiia and Partington (2019) argue, spokespersons' discourse is a genre regularly employing forced lexical priming (Duguid, 2009) that refers to the deliberate and frequent repetition of certain lexical forms to “flood” the discourse with strategically framed messages. Such forced priming of collocational patterns involving “virus” and “pandemic” by the CMFA spokespersons was strategically employed and intended to ensure that both the media in press conference contexts and wider audiences internalize China's official stances.

## 4 Discussion

Discourse, as a form of social practice, is intrinsically tied to power and ideology. “In every society the production of discourse is at once controlled, selected, organized and redistributed by a certain number of procedures … to gain mastery over its chance events” (Foucault, 1981, p. 52).

To further explore how power and ideology manifest through discourse, van Dijk (1998, p. 6) emphasizes discourse's critical role in shaping ideological structures and argues that examining the regular patterns within discursive practices is essential to understanding how ideologies emerge, evolve, and persist. Specifically, van Dijk's (1998, p. 267) Ideological Square model provides a robust analytical framework by outlining a discursive structure of positive self-presentation and negative other-presentation. Importantly, the Ideological Square model is characterized by two core features: firstly, it emphasizes the group-based nature of ideology; secondly, ideology is inherently self-serving (van Dijk, 1998, p. 68–69).

It is noteworthy that as a key element of China's diplomatic practices, spokespersons' discourse is ideologically charged and strategically constructed. The repeated discursive choices–such as the collocational patterns of “virus” and “pandemic”—reveal underlying ideological positions influenced by China's geopolitical relations with the West and by its diplomatic philosophy rooted in distinct cultural and historical contexts.

### 4.1 Geopolitical relations and diplomatic ideology in the spokespersons' discourse

In its diplomatic discourse, China highlights its guiding principles in developing relations with other countries—principles rooted in peace, development, cooperation, and mutual benefit. Particularly to the US, the Chinese officials, from Chinese President to the CMFA spokespersons, consistently highlight the importance of the China-US relations to the stability and prosperity to the world and seek for cooperations rather than competitions. This commitment is most evident in its promotion of the diplomatic concept of building “a community with a shared future for mankind” (Hu, 2012; Xi, 2015; Nathan and Zhang, 2022).

However, China and the US have in recent years experienced geopolitical tensions and clashes of interest, particularly in the context where the US politicians started trade wars, imposed restrictions on Chinese enterprises, and portrayed China as both a threat and a competitor in their public rhetoric. For example, the Trump administration (2017–2021) reinforced this stance by officially designating China as a “strategic competitor” in documents such as The National Security Strategy Report, The National Defense Strategy Report, and The Nuclear Posture Review, emphasizing that the competition between the two countries is strategic, long-term, and all-encompassing. Such geopolitical relation tension between the US-led Western countries and China was reflected in the CMFA spokespersons' discourse in the context of the global COVID-19 pandemic.

During the pandemic, the US-led Western countries continuously demonized China, propagated the “Chinese virus” narrative, questioned China's pandemic measures, and accused China of using its assistance to other nations for political gain. In response, the CMFA spokespersons drew on communicative discursive strategies to highlight the scientific basis of the novel coronavirus's origin, showcasing China's collective efforts and determination in combating the pandemic. They also emphasized China's promotion of international collaboration in pandemic prevention, reinforcing the diplomatic principle of “a community with a shared future for mankind”.

Interestingly, they simultaneously utilized a juxtapositional discursive strategy of presenting China, together with some other countries, as the positive majority (frequency accounting for 52.93%) while portraying the US and the UK's media BBC as the negative minority (frequency accounting for only 6.09%) to further justify the rationality of China's policies. This aligns with Danziger and Schreiber's (2021) argument that, similar to individual interaction, a country's international communication involves projecting its values and norms through discourse to win support in the global community. This result also provides partial evidence for van Dijk's Ideological Square model, while also expanding the conventional boundaries of “Self” and “Others”, positioning the “Self” among the positive majority and the “Others” among the negative minority.

Meanwhile, China also advocates for a new model of international relations founded on mutual respect, fairness and justice. Regarding the unjust allegations without respect, China has tried responses with a spirit of struggle. This is manifested in Chinese President Xi Jinping's speech at the opening ceremony of a training class for officials at the Party School of the CPC Central Committee September 1, 2021, which states,

“It is unrealistic to always want peaceful days and avoid struggle. We must abandon illusions, be brave in struggle, stand firm on principle, and never yield an inch with unprecedented determination and resolve when defending the sovereignty, security, and development interests of our country.”

This explains that the spokespersons actively participated in a tit-for-tat struggle on the diplomatic frontline by adopting offensive discourse strategies in response to the smears and accusations against China by the US-led Western countries regarding the virus and the pandemic. Motivated by this ideological stance, they resolutely rebutted unfounded allegations and criticized the ineffective and irresponsible handling of the pandemic by the Western countries. This is reflected in Poh and Li (2017) finding that both the academic and political fields have increasingly acknowledged China's steady strengthening of its voice on matters concerning its diplomatic and security interests in recent years.

### 4.2 Traditional Chinese culture and contemporary China's diplomacy

In the context of the CMFA spokespersons' responses, the diverse representations of other countries' images also find their roots in traditional Chinese culture, particularly Confucianism, which has long shaped China's political, social, and ethical values and provided a foundational framework for China's contemporary diplomatic discourse (Xing, 2015; Lajčiak, 2017). As a classic in Confucianism, Analects of Confucius–Xian Wen contains a philosophical concept that influences the construction of interpersonal and, by extension, international relationships. The concept can be perceived in the dialogue between Confucius and his disciples:

Someone asked, “What about repaying evil with kindness?” Confucius said, “How then will you repay kindness? Repay evil with justice, and repay kindness with kindness”.

China's diplomatic orientation in dealing with international relations is deeply rooted in this traditional philosophy. As an illustration, when he was invited to give a speech in Berlin on 28^th^ March, 2014, the Chinese President Xi Jinping remarked that, “[W]e do not provoke trouble, but we are not afraid of it. We will firmly defend China's legitimate rights and interests”, which embodies well the idea that “repaying evil with justice and repaying kindness with kindness”.

As an alternative to Western approaches, China advocates resolving conflicts through dialogue, pursues mutually beneficial solutions via peaceful means, and promotes the establishment of a new type of international relations. However, China remains unswerving in defending its national interests and will spare no efforts to counter any attempts to smear or suppress it in its diplomatic practices. During the COVID-19 pandemic, CMFA spokespersons drew on various discursive strategies in regular press conferences to address misunderstandings and counter negative representations of China driven by Western countries' zero-sum mentality. By contrast, toward friendly nations like Russia, the spokespersons actively fostered positive representations in their diplomatic discourse.

## 5 Conclusion

By a corpus- driven analysis of the collocational characteristics of the terms “病毒” and “疫情” in the CMFA spokespersons' responses during the regular press conferences, this paper tried to identify the discursive strategies by the spokespersons. It is found that the spokespersons employed communicative discursive strategies to highlight the scientific nature of virus origin-tracing and promote international cooperation against COVID-19 as a global challenge and at the same time they adopted offensive discursive strategies to refute smears and accusations from the US-led Western countries by criticizing their ineffective policies against Corvid-19 and their hegemonic intervention in the internal affairs of other countries. It is argued that the collocational characteristics of “病毒” and “疫情” are closely tied to ideological factors underpinning China's diplomacy, which are a result of the joint influence of China's geopolitical dynamics with the West and the impact of traditional Chinese culture on its diplomatic practices.

This study contributes to the expanding body of research on corpus-driven analyses of diplomatic discourse, particularly in the Chinese context and the identification of the juxtapositional discursive strategy in this study also helps extend the current Ideology Square model as a framework for discourse analysis. However, further research is needed to broaden the scope of investigation. For instance, a comparative analysis of the collocational patterns of COVID-19-related terms in the diplomatic discourse of China with that of the US or with Western media discourse could provide more valuable insights. It would also be interesting to further examine the discursive strategies in the diplomatic discourse of China and the US from a diachronic perspective.

## Data Availability

Publicly available datasets were analyzed in this study. This data can be found at: https://github.com/lt0806/Data.git.
